# Intact cell lipidomics using the Bruker MBT lipid Xtract assay allows the rapid detection of glycosyl-inositol-phospho-ceramides from *Aspergillus fumigatus*†

**DOI:** 10.1039/d4mo00030g

**Published:** 2024-03-28

**Authors:** Aishani Chakraborty, Leila Alsharqi, Markus Kostrzewa, Darius Armstrong-James, Gerald Larrouy-Maumus

**Affiliations:** a Centre for Bacterial Resistance Biology, Department of Life Sciences, Faculty of Natural Sciences, Imperial College London London SW7 2AZ UK g.larrouy-maumus@imperial.ac.uk; b Centre for Bacterial Resistance Biology, Department of Infectious Diseases, Faculty of Medicine, Imperial College London London SW7 2AZ UK d.armstrong@imperial.ac.uk; c Bruker Daltonik GmbH Bremen 28359 Germany

## Abstract

Glycosyl-inositol-phospho-ceramides (GIPCs) or glycosylphosphatidylinositol-anchored fungal polysaccharides are major lipids in plant and fungal plasma membranes and play an important role in stress adaption. However, their analysis remains challenging due to the multiple steps involved in their extraction and purification prior to mass spectrometry analysis. To address this challenge, we report here a novel simplified method to identify GIPCs from *Aspergillus fumigatus* using the new Bruker MBT lipid Xtract assay. *A. fumigatus* reference strains and clinical isolates were cultured, harvested, heat-inactivated and suspended in double-distilled water. A fraction of this fungal preparation was then dried in a microtube, mixed with an MBT lipid Xtract matrix (Bruker Daltonik, Germany) and loaded onto a MALDI target plate. Analysis was performed using a Bruker MALDI Biotyper Sirius system in the linear negative ion mode. Mass spectra were scanned from *m*/*z* 700 to *m*/*z* 2 000. MALDI-TOF MS analysis of cultured fungi showed a clear signature of GIPCs in *Aspergillus fumigatus* reference strains and clinical isolates. Here, we have demonstrated that routine MALDI-TOF in the linear negative ion mode combined with the MBT lipid Xtract is able to detect *Aspergillus fumigatus* GIPCs.

## Abstract 2

Glycosyl-inositol-phospho-ceramides (GIPCs) or glycosylphosphatidylinositol-anchored fungal polysaccharides are major lipids in plant and fungal plasma membranes and play an important role in stress adaption.

## Graphical abstract

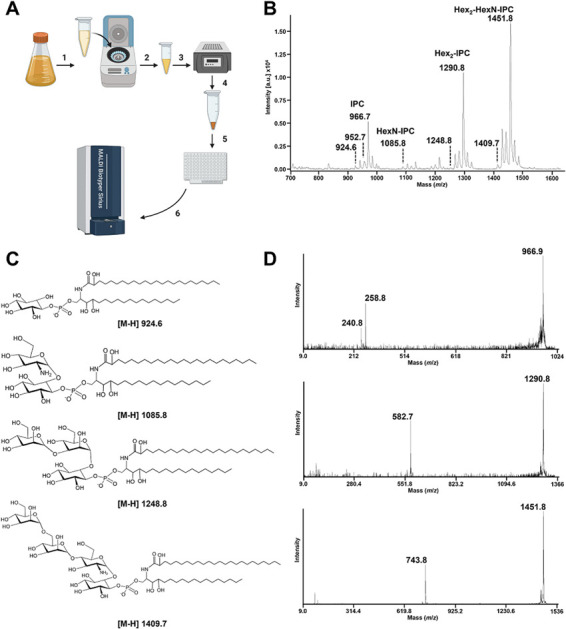

## Introduction

*Aspergillus fumigatus* was recently identified as one of the four World Health Organisation's (WHO) critical priority fungal pathogens. It is an opportunistic pathogen that does not cause infections in healthy individuals but leads to severe and sometimes fatal infections in immunocompromised patients.^1^ Furthermore, in people living with cystic fibrosis (CF), almost 8–10% experience allergic bronchopulmonary aspergillosis that is caused by colonisation and subsequent allergic responses to *A. fumigatus*. A key feature of the filamentous fungus *A. fumigatus* is a cell wall extremely rich in polysaccharides and glycolipids such as galactomannan (GM) and glycosylinositolphosphoceramides (GIPCs), respectively.^1–3^ GM is a unique fungal polysaccharide containing a tetramannoside repeat unit that can be membrane bound through a glycosylphosphatidylinositol (GPI)-anchor covalently linked to -1,3-glucans in the cell wall, or released in the culture medium as a free polymer.^3,4^ GIPCs are involved in cell growth and fungal–host interactions^3–7^ and are acidic glycosphingolipids containing a phosphodiester linkage between inositol and a ceramide moiety composed of phytosphingosine associated with a saturated long chain (C18–26) fatty acid with or without a hydroxyl group in position 2, and a variable carbohydrate moiety.^2,5^

Great efforts have been made to establish the structure of GM,^1,3^ but the analysis of GIPCs and their structural characterization using mass spectrometry remain highly challenging and time-consuming.^5–7^ Indeed, multiple enrichment steps such as organics solvent extractions, partitions and hydrophobic interaction chromatography are required for purification of GIPCs prior to MALDI-TOF mass spectrometry.^8–12^ MALDI-TOF is a “soft-ionization” technique which ionizes large biomolecules while keeping the original structure intact.^13^ It produces rapid results, is easy to use and is cost-efficient.^14^ Recently, by exploiting the potential of “soft-ionization” combined with the appropriate matrix and sample preparation, MALDI-TOF has been used for the analysis and the characterization of complex lipids from bacteria such as lipid A directly from whole bacteria without the need for fractionation or purification.^15–22^ Indeed, we and others have developed methods to facilitate lipid micro-extraction prior to MALDI-TOF MS or by direct detection of lipid A molecules and their modifications in bacteria using a routine MALDI-TOF mass spectrometer.^16,22–34^

However, to our knowledge, such an approach has not yet been used in the identification and characterization of *A. fumigatus* GIPCs.

The new Bruker MBT lipid Xtract assay was originally developed for the detection of lipid A and the modifications such as phosphoethanolamine and l-aminoarabinose that confer resistance to polymyxins.^22^ Here, we have taken an intact cell lipidomic approach using the Bruker MBT lipid Xtract assay and used it for the rapid detection of GIPCs found in the *A. fumigatus* cell wall using MALDI-TOF MS in negative ion mode.

## Materials and methods

### *Aspergillus fumigatus* isolates and azole susceptibility determination

Nineteen *Aspergillus fumigatus* strains were provided by the Royal Brompton Hospital. Fungi were grown in liquids in the Sabaraud dextrose medium at 30 °C overnight. To determine the strain azole susceptibility profile, a 4-well VIPcheck^TM^ plate (Balis Laboratorium, Boven–Leeuwen, The Netherlands) was used to screen all isolates used in this study. The VIPcheck™ plate consists of a four-well plate, containing RPMI agar supplemented with three medical azoles, itraconazole (4 mg L^−1^), voriconazole (4 mg L^−1^) and posaconazole (0.5 mg L^−1^), and an azole-free growth control. Conidial harvest suspensions were used. In addition, any azole-resistant isolate was confirmed with a standard microbroth dilution procedure according to the EUCAST reference guideline.^35^

For MALDI analysis, 1 mL of the cultures were transferred into a 1.5 mL microtube prior to heat-inactivation for 1 h at 90 °C in a water-bath. Cultures were analysed in biological duplicates.

### Sample preparation

The heat-inactivated fungi were washed three times with 0.5 mL of double distilled water at 17 000 × g for 5 min. Fungi were finally suspended at a concentration of McFarland 40 in ddH_2_O and the suspension vortexed for 30 Section. 2 μL of the samples were then added to new microtubes that were placed for 5 minutes with the lid open in a heat block set at 98 °C to concentrate the sample and create a film at the bottom of the microtube. The dried pellet was resuspended in 2 μL of the MBT Lipid Xtract Matrix (Part No. 1889112, Bruker Daltonik, Germany) by pipetting up and down while gently scraping the walls. The samples were loaded onto a MBT MALDI Biotarget 96 plate (Part-No. 1840375, Bruker Daltonik, Germany). For external calibration, 0.5 μL of a calibration peptide was loaded along with 0.5 μL of the calibration matrix (peptide calibration standard II, Bruker Daltonik, Germany).

## MALDI-TOF MS and data analysis

MS analyses were performed using a MALDI Biotyper Sirius system (Bruker Daltonik, Germany) within the range of *m*/*z* 700 to 2 000 employing the linear negative-ion mode (laser intensity 75%, ion source 1  =  15.00 kV, ion source 2  =  8.98 kV, lens = 3.00 kV, detector voltage  =  2652 V, and pulsed ion extraction  =  150 ns). Each spectrum correspond to an ion accumulation of 500–1 000 laser shots randomly distributed on the spot. The mass profiles and spectra were processed with default parameters using the FlexAnalysis v.3.4 software (Bruker Daltonik, Germany). Data pre-processing was performed as described previously.^36,37^

Assignments were based on the MS/MS fragmentation profile acquired using a 4 800 Proteomics Analyzer (with TOF-TOF Optics, Applied Biosystems, plate: 384 Opti-TOF 123 mm × 84 mm AB Sciex NC0318050, 1016629) using the reflectron negative-ion mode. Samples were analyzed operating at 20 kV in the negative ion mode. MS/MS mass spectrometry data were analyzed using Data Explorer version 4.9 from Applied Biosystems. All experiments were performed in biological and technical duplicates.

## Results and discussion

**Fig. 1:**
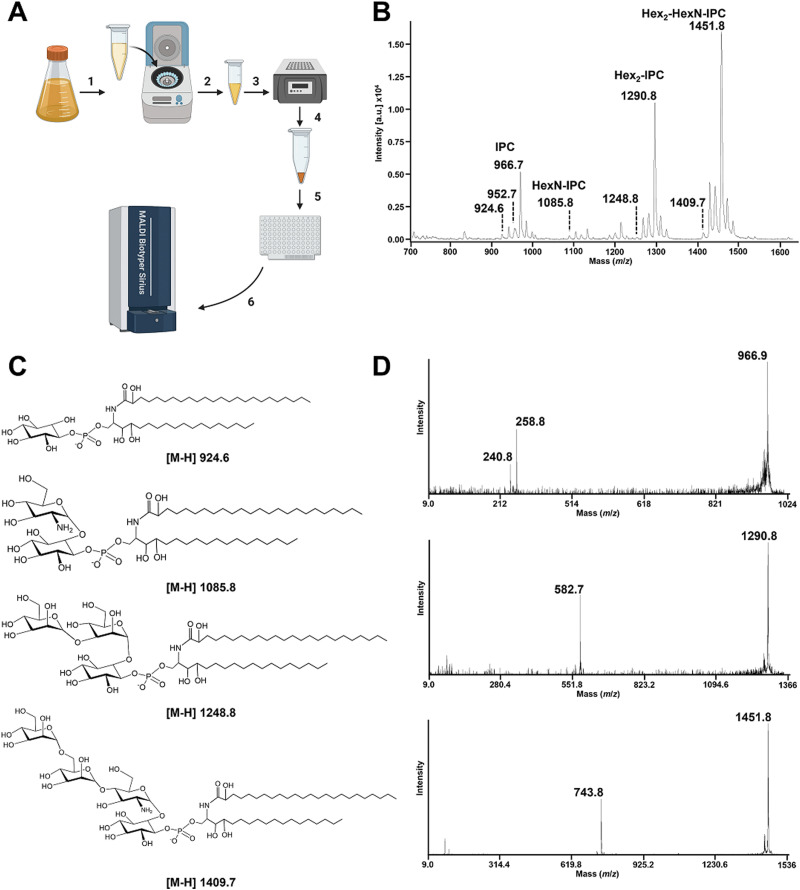
Rapid detection of *A. fumigatus* GIPCs by MALDI-TOF mass spectrometry in the negative ion mode. (A) Schematic representation of the simplified workflow to obtain a rapid GIPC fingerprint from *A. fumigatus*. Cultured *A. fumigatus* was pelleted in a microtube, (1) and solubilized at a defined concentration such as McFarland 20 to 50 (2). 2 μL of the prepared fungal solution was dried using a heat-block for 5 min set at 98 °C (3). 2 μL of the MBT lipid Xtract matrix was then used to resuspend the film formed by the dried fungi (4) and was deposited on the MALDI target plate (5). Mass spectra were acquired using a MALDI Biotyper Sirius system within the range *m*/*z* 700 to *m*/*z* 2 000 operating in linear negative ion mode. (B) MALDI-TOF negative linear ion mode mass spectra of the *A. fumigatus* CEA102 reference strain. (C) Structure of the GIPCs detected in the negative ion mode. (D) MALDI-TOF MS/MS mass spectra of the [M–H]- ions at *m*/*z* 966.9, *m*/*z* 1290.8, and *m*/*z* 1451.8. The product ions at *m*/*z* 258.8 and 240.8 correspond to the polar head group assigned as inositolphosphate and inositolphosphate minus a molecule of water, respectively. The product ions at *m*/*z* 582.7 and *m*/*z* 743.8 corresponding to the [M–H]^−^ ions at *m*/*z* 1290.8 and *m*/*z* 1451.8, respectively, are assigned to [hexose-hexose-inositol-monophosphate]- and [hexose-hexose-hexosamine-inositol-monophosphate]-.

GIPCs are negatively charged molecules; therefore, we used the MALDI Biotyper sirius® system in linear negative ion mode (Fig. 1A). Lipid assignments were based on the MS/MS profiles with the same volume of the original samples that was used for MS analyses, alongside the LIPID MAPS database (http://www.lipidmaps.org/) and data published in the literature.^5,6,38–40^

In the reference strain *A. fumigatus* CEA102, the linear negative ion mass spectrum (Fig. 1B) is dominated by a set of peaks at *m*/*z* 924.6, *m*/*z* 1085.8, *m*/*z* 1290.8, and *m*/*z* 1451.8. The MS/MS analysis of the ion at *m*/*z* 924.6 led to two major fragments at *m*/*z* 240.8 and *m*/*z* 258.8 assigned to [inositol-1,2-cyclicphosphate]- and [inositol-phosphate]-, respectively (Fig. 1D). The absence of fragment ions arising from acyl chains is the characteristic of the presence of a ceramide group.^41^ This confirmed that the [M−H]^−^ pseudomolecular ion at *m*/*z* 924.6 is assigned to an inositolphosphoceramide (IPC) composed of the C18-phytoshingosine and 2-monohydroxylated-C24 : 0 fatty acids as described previously.^7,38^ The ions [M–H]^−^ at *m*/*z* 938.7, *m*/*z* 952.7 and *m*/*z* 966.7 correspond respectively to different forms of the ceramide where a hydroxyl group in the fatty acid has been replaced by hydrogen, and the addition of one, two or three methylene groups in the aliphatic chain.

The series of [M−H]^−^ pseudomolecular ions observed at *m*/*z* 1085.8, *m*/*z* 1290.8, and *m*/*z* 1451.8 correspond to an increase of a combination of hexose and hexosamine from the [M–H]^−^ pseudomolecular ion at *m*/*z* 924.6 and the dominant peak at *m*/*z* 966.7 (Fig. 1B). This is confirmed by MS/MS where the fragmentation of the ion at *m*/*z* 1290.8 produced a daughter ion at *m*/*z* 582.7 assigned to [hexose-hexose-inositol-monophosphate]-, the fragmentation of the ion at *m*/*z* 1451.8 produced a daughter ion at *m*/*z* 743.8 assigned to [hexose-hexose-hexosamine-inositol-monophosphate]-. A similar profile and assignment have been reported in the literature^5^ upon organic phase extraction of *Aspergillus fumigatus* suggesting that the simplified protocol used here is adapted for apolar GIPCs.

**Fig. 2:**
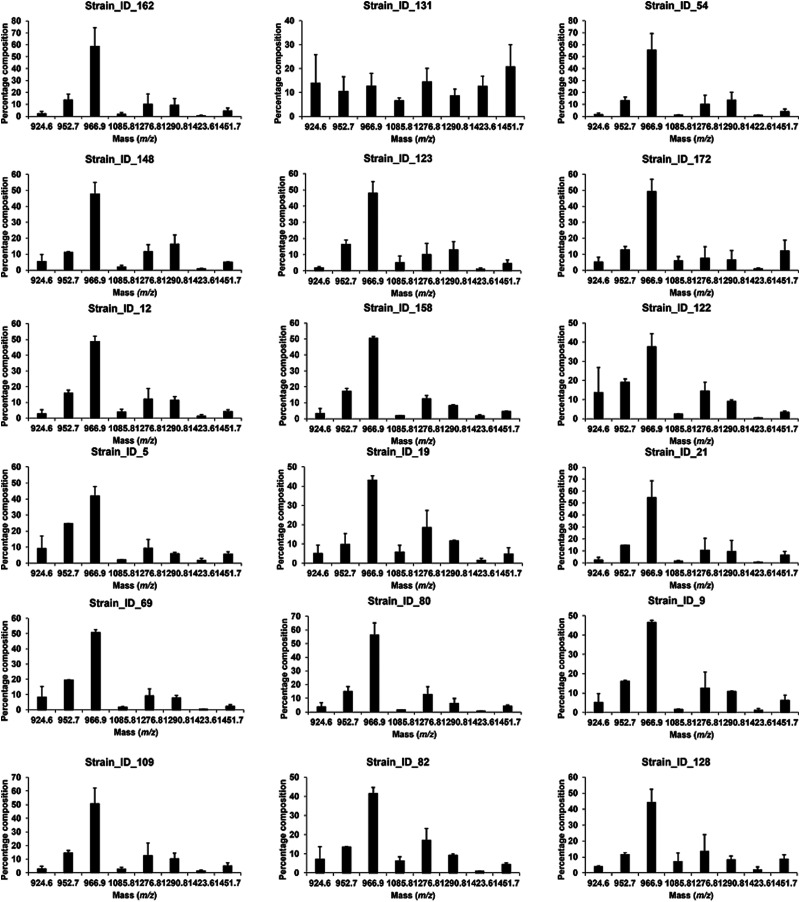
Bar graphs displaying the intensities of the key GIPCs *A. fumigatus* clinical isolates.

To test if this novel workflow can be applied to *A. fumigatus* clinical isolates, a collection of 18 strains was submitted to the workflow presented in Fig. 1A. As seen in Fig. 2, a similar GIPC profile to the one recorded for the CEA102 reference strain was recorded for all strains except strain 131, with the major GIPC detected at *m*/*z* 966.9 assigned to inositolphosphoceramide.

We report here on a simple and rapid assay to detect fungal GIPCs from *A. fumigatus* using a routine MALDI mass spectrometer in the negative ion mode using the newly developed MBT lipid Xtract assay kit (Bruker Daltonik, Germany). One limitation of the study is the limited numbers of strains used and their diversity. A more diverse collection of strains and fungi, ideally involving different laboratories, would determine the robustness of the approach. Finally, integration of the MALDI data acquired with machine learning algorithms has the potential to discriminate and classify isolates for their drug susceptibility and virulence. In addition, this approach can be used for the discovery of discriminatory species-specific markers, such as the abundance of the differently glycosylated GIPCs; the saccharide moiety composition could be then used as a discrimination marker. A substantial number of reference strains and clinical isolates would be needed to test this potential application. This approach could also be used for drug screening assays which target enzymes involved in GIPC biosynthesis and potentially decrease the viability or virulence of *A. fumigatus*. This approach has been employed for the discovery of natural compounds that target the phosphoethanolaminetransferase MCR-1 in *E. coli* leading to colistin resistance.^42^

In addition, the idea to use this approach to clinical diagnostics specimens has some potential. However, it would require ethical approval to use human biofluids and regulatory approval following clinical validation.

The novel and easy to implement approach reported here for rapidly fingerprinting GIPCs from *A. fumigatus* has a wide range of applications (Table 1).

**Table 1:** List of strains used in this study

Strain number	Origin of the strains	Drug profile
CEA102	Reference	Susceptible
162	CF/ABPA	Susceptible
148	Asthma/ABPA	Susceptible
12	Sarcoid/CPA	Susceptible
5	Bronchiectasis/ABPA	Susceptible
69	Bronchiectasis/CPA	Susceptible
109	Bronchiectasis/ABPA	Susceptible
131	CF/Asp colonisation	Resistant
123	CF/Asp Bronchitis	Resistant
158	CF/Asp bronchitis	Resistant
19	CF/Asp bronchitis	Resistant
80	CF/Asp bronchitis	Resistant
82	CF/Asp bronchitis	Resistant
54	CF/Asp Bronchitis	Resistant
172	CF/CPA	Resistant
122	Bronchiectasis/CPA	Resistant
21	Asthma/ABPA	Resistant
9	Sarcoid/CPA	Resistant
128	Sarcoid/CPA	Resistant

## Data availability

All data generated during this study are included in this published article.

## Author contributions

Study conception, design, and coordination: AC, LA, MK, DAJ and GL-M. Sample analysis: AC and LA. Experimental design and implementation: AC, LA, MK, DAJ and GL-M. Data analysis and writing the manuscript: all authors.

## Conflicts of interest

MK is an employee of Bruker Daltonics GmbH & Co. KG, the manufacturer of the MALDI-TOF MS system used in this study, MBT Lipid Xtract™ Kit. The remaining authors have no conflicts of interest to declare. There are no conflicts to declare.

## Supplementary Material

MO-020-D4MO00030G-s001

MO-020-D4MO00030G-s002
