# Supplementary material for: Intact cell lipidomics using the Bruker MBT lipid Xtract assay allows the rapid detection of glycosyl-inositol-phospho-ceramides from Aspergillus fumigatus
Source: Mol Omics. 2024 Mar 28;20(6):390–6. doi: 10.1039/d4mo00030g (PMC11228930; doi:10.1039/d4mo00030g)
Supplement: MO-020-D4MO00030G-s001 [file MO-020-D4MO00030G-s001.pdf]

Electronic Supplementary Material (ESI) for Molecular Omics.
This journal is © The Royal Society of Chemistry 2024

Electronic supplementary information (ESI) file (raw MS data)

Spotset_2

**Spot**	**Strain**
B9	CEA102
B6	123
A8	21
A1	172
A3	12
A5	82
B4	54
A6	80
B3	148
B1	162
B10	19
A7	128
A11	5
A10	109
B2	158
B5	9
B7	69
A4	122
A12	131

Spotset_1

**Spot**	**Strain**
D11	CEA10
E3	123
E4	21
E5	172
E6	12
E7	82
E8	54
E9	80
E10	148
E11	162
E12	19
F1	128
F2	5
F3	109
F4	158
F5	9
F7	69
F8	122
F9	131
