# Supplementary material for: Intact cell lipidomics using the Bruker MBT lipid Xtract assay allows the rapid detection of glycosyl-inositol-phospho-ceramides from Aspergillus fumigatus
Source: Mol Omics. 2024 Mar 28;20(6):390–6. doi: 10.1039/d4mo00030g (PMC11228930; doi:10.1039/d4mo00030g)
Supplement: MO-020-D4MO00030G-s002 [file MO-020-D4MO00030G-s002.zip › ESI_raw_data/Electronic supplementary information file .docx]

Electronic supplementary information (ESI) file (raw MS data)

| **Spotset_2** | |  | **Spotset_1** | |
| --- | --- | --- | --- | --- |
|  |  |  |  |  |
| **Spot** | **Strain** |  | **Spot** | **Strain** |
| B9 | CEA102 |  | D11 | CEA10 |
| B6 | 123 |  | E3 | 123 |
| A8 | 21 |  | E4 | 21 |
| A1 | 172 |  | E5 | 172 |
| A3 | 12 |  | E6 | 12 |
| A5 | 82 |  | E7 | 82 |
| B4 | 54 |  | E8 | 54 |
| A6 | 80 |  | E9 | 80 |
| B3 | 148 |  | E10 | 148 |
| B1 | 162 |  | E11 | 162 |
| B10 | 19 |  | E12 | 19 |
| A7 | 128 |  | F1 | 128 |
| A11 | 5 |  | F2 | 5 |
| A10 | 109 |  | F3 | 109 |
| B2 | 158 |  | F4 | 158 |
| B5 | 9 |  | F5 | 9 |
| B7 | 69 |  | F7 | 69 |
| A4 | 122 |  | F8 | 122 |
| A12 | 131 |  | F9 | 131 |
